# Structuring, reuse and analysis of electronic dental data using the Oral Health and Disease Ontology

**DOI:** 10.1186/s13326-020-00222-0

**Published:** 2020-08-20

**Authors:** William D. Duncan, Thankam Thyvalikakath, Melissa Haendel, Carlo Torniai, Pedro Hernandez, Mei Song, Amit Acharya, Daniel J. Caplan, Titus Schleyer, Alan Ruttenberg

**Affiliations:** 1 National Center for Ontological Research, Buffalo, NY USA; 2 Center for Biomedical Informatics, Regenstrief institute, Inc., Indianapolis, IN USA; 3 Dental Informatics Core, Indiana University School of Dentistry, Indianapolis, IN USA; 4 Translational and Integrative Sciences Lab, Oregon State University, Corvallis, OR USA; 5 Domino Data Lab, San Francisco, CA USA; 6 Reparto Universitario, San Juan, PR USA; 7 Magee-Women’s Research Institute, Pittsburgh, PA USA; 8 Marshfield Clinic Research Institute, Marshfield, WI USA; 9 University of Iowa College of Dentistry, Iowa City, IA USA; 10 Indiana University School of Medicine, Indianapolis, IN USA; 11 School of Dental Medicine, State University of New York at Buffalo, Buffalo, NY USA

**Keywords:** Ontology, Dental health, Informatics, Electronic heath record, OWL, SPARQL

## Abstract

**Background:**

A key challenge for improving the quality of health care is to be able to use a common framework to work with patient information acquired in any of the health and life science disciplines. Patient information collected during dental care exposes many of the challenges that confront a wider scale approach. For example, to improve the quality of dental care, we must be able to collect and analyze data about dental procedures from multiple practices. However, a number of challenges make doing so difficult. First, dental electronic health record (EHR) information is often stored in complex relational databases that are poorly documented. Second, there is not a commonly accepted and implemented database schema for dental EHR systems. Third, integrative work that attempts to bridge dentistry and other settings in healthcare is made difficult by the disconnect between representations of medical information within dental and other disciplines’ EHR systems. As dentistry increasingly concerns itself with the general health of a patient, for example in increased efforts to monitor heart health and systemic disease, the impact of this disconnect becomes more and more severe.

To demonstrate how to address these problems, we have developed the open-source *Oral Health and Disease Ontology* (OHD) and our instance-based representation as a framework for dental and medical health care information. We envision a time when medical record systems use a common data back end that would make interoperating trivial and obviate the need for a dedicated messaging framework to move data between systems.

The OHD is not yet complete. It includes enough to be useful and to demonstrate how it is constructed. We demonstrate its utility in an analysis of longevity of dental restorations. Our first narrow use case provides a prototype, and is intended demonstrate a prospective design for a principled data backend that can be used consistently and encompass both dental and medical information in a single framework.

**Results:**

The OHD contains over 1900 classes and 59 relationships. Most of the classes and relationships were imported from existing *OBO Foundry* ontologies. Using the LSW2 (*LISP Semantic Web*) software library, we translated data from a dental practice’s EHR system into a corresponding *Web Ontology Language* (OWL) representation based on the OHD framework. The OWL representation was then loaded into a triple store, and as a proof of concept, we addressed a question of clinical relevance – a survival analysis of the longevity of resin filling restorations. We provide queries using SPARQL and statistical analysis code in R to demonstrate how to perform clinical research using a framework such as the OHD, and we compare our results with previous studies.

**Conclusions:**

This proof-of-concept project translated data from a single practice. By using dental practice data, we demonstrate that the OHD and the instance-based approach are sufficient to represent data generated in real-world, routine clinical settings. While the OHD is applicable to integration of data from multiple practices with different dental EHR systems, we intend our work to be understood as a prospective design for EHR data storage that would simplify medical informatics. The system has well-understood semantics because of our use of BFO-based realist ontology and its representation in OWL. The data model is a well-defined web standard.

## Background

A key challenge for improving the quality of healthcare is to be able to use a common framework to work with patient information acquired in any of the health and life science disciplines. The patient information collected during dental care exposes many of the challenges that confront a wider scale approach. Within dentistry, a key aspect for improving the quality of care is the ability to collect and analyze data about oral health conditions and procedures, such as the longevity of fillings, the frequency of patient checkups, and incidence of tooth loss. Recent reports estimate that 73.8% of solo practitioners and 78.7% of group practitioners in the U.S. use a computer to manage some, and 14.3 and 15.9%, respectively, all patient information on a computer [1] . In consequence, we now have the opportunity to study dental health services and perform outcomes research using large amounts of secondary data obtained from geographically dispersed dental practices [2].

Large secondary datasets could help us more easily study diseases in a sizable samples with increased statistical power, track patients for an extended period of time, provide valid and representative samples, supply correlates not commonly collected in an oral health setting, collect data in real time and ascertain potential confounders [2].

Analyzing data from electronic health records (EHR), however, presents a number of challenges. First, dental EHR information is often stored in relational databases that are poorly documented and have complex relations between tables. This makes extracting and analyzing data from even a single practice’s system difficult. Second, dental EHR database schemas vary depending on the vendor who developed the system. This adds difficulty when integrating data from multiple practices. Third, information is not always encoded in the same way. For example, a tooth encoded as number (e.g., tooth ‘6’) or as a character array in which the index position of a character represents the tooth (e.g., the ‘Y’ in the character array ‘NNNNNYNNNNNNNNNNNNNNNNNNNNNNNNNN’ represents a right upper secondary canine tooth, i.e., tooth ‘6’). Last, dental EHR systems are typically only loosely specified. So, outside of a common core of structures for the oral cavity and its parts, there is wide variation in how information such as specific types of materials, details of methods, instruments, and general patient health is represented. Much of this information is either semi-or unstructured text. While we focus here on dental EHRs, these same problems are endemic in other EHR systems.

To demonstrate how to address some of these problems, we have developed the *Oral Health and Disease Ontology* (OHD) as a common framework for representing dental health information embedded in a larger framework adequate to accommodate structured representation that goes beyond that in current dental EHR systems and extends into general medicine. The OHD contains terms for representing anatomical structures (e.g., distal surface of tooth), dental procedures (e.g., tooth extraction), and oral conditions (e.g., caries), as well as relations between terms (e.g., distal surface is part of tooth). The OHD’s structure provides a common representation of the entities that EHR data is about, without being designed in a way that unintentionally limits it to *only* dental health data. This makes it possible to use the OHD as framework for integrating inhomogeneous data from disparate database systems and support representations for future systems. Using the OHD’s terms and relations, information from multiple dental EHRs can now be translated into OWL 2 [3] statements, stored in a semantic database or triple store, and queried using SPARQL [4] to extract information for analysis.

As a proof of concept, we have translated dental EHR data from a single dental practice, and performed a survival analysis of the longevity of resin filling restorations. The proof of concept demonstrates both aspects of the OHD that are specific to dentistry (e.g. teeth, restorations and other procedures) as well as aspects that would remain unchanged in a general medical context, such as demographic correlates. The output of this analysis is discussed in the *Results* section. In the *Discussion* section we describe the potential for wider application.

## Related work

The work in this paper expands upon previous work developing the OHD [5, 6], and provides a more detailed explication of the OHD’s structure. It differs from previous ontology work, such as PeriO [7] and BigMouth [8], in two respects.

First, it focuses on the domain of dental anatomy and procedures rather than genomic information. Second, the OHD’s use of the Basic Formal Ontology and Ontology of Biomedical Investigations as an upper-level framework sets the stage for seamlessly extending it to general medical information. Moreover, the OHD is not a data repository, such as BigMouth [8], but a semantic framework for representing data that may be used in the design of repositories – such as our semantic technology-based repository of information translated from (for now) a single dental practice.

We considered using SNOMED and its dental subset SNODENT, but there are problems that make these standards, at the moment, unusable for our purposes. First, their licenses restrict modification of substantial parts of the standard. This prevents us from reorganizing content according to realist principles, adding definitions, or adding or correcting axioms. Not all countries license to use SNOMED, and this would prevent our work from being replicable worldwide.

Second, there are serious quality issues with SNOMED, and SNODENT in particular [9]. A major issue is the question of ontological commitment – what terms mean. The vast majority of terms in SNODENT and SNOMED come without textual definitions [10], and the question of what SNOMED terms actually represent is still up for debate.

Third, use of these terminologies typically is within a layered framework that brings unnecessarily complication [11]. In common usage these resources are *bound* to data models of medical records [12]. That means that one needs to separately understand the data models and the ontology. By contrast, in our approach the ingredients for a representation are simple – an OWL ontology and high-quality SPARQL, OWL and RDF W3C specifications. Those logic-based specifications are substantially clearer than HL7 specifications.

The Open Biological and Biomedical Ontology (OBO) Foundry approach is to have, for any given class, a single identifier, if necessary coordinating with developers of other ontologies. The realism-based approach emphasizes that classes are collections of instances, that the instances are things in the world, and that documentation should make clear what those instances are. The OHD and the semantic technologies used to implement the ontology make it relatively easy to merge data. The data is just added together, untransformed. It is possible to do this, in theory, because all parts of the representation are clearly understood, the types of entities are shared, and the choice to represent particulars using the standard methods provided by semantic web standards allow for little creativity in how concrete representations are constructed. Because our focus is on showing how a unified representation system works, we consider out of scope general methods for harmonizing or interchanging data with different representations, as is the focus of HL7.

Recently, authors AR and WD have started participating in the review and development of SNODENT. It is entirely possible that in the future that SNOMED and SNODENT might be used in the same manner that we use OHD here. The OHD and the source code used for translation and analysis are available in full at https://github.com/oral-health-and-disease-ontologies/ohd-ontology and in part in the Additional file 1.

## Methods

### Ontology development

The OHD was developed in a collaborative effort between dental researchers, practicing dentists, statisticians, informatics experts, and ontologists. Our first task was to identify which dental entities would be represented. To guide this process, we developed a set of research questions. For example, for the research question, “What is the time from one restoration to its replacement on the same tooth?”, we determined that we would need to represent restoration procedures, the dates of the procedures, patients, patients’ teeth, surfaces of teeth, and the restoration materials used to restore teeth. We provide the list of driving research questions in Additional file 1.

Once our domain of focus was identified, our next step was to catalog the terms^1^ we would need in the ontology. We imported the Basic Formal Ontology (BFO) and the Ontology for General Medical Science (OGMS) as a whole and otherwise extracted terms from existing OBO Foundry ontologies that represented entities relevant to our dental health domain using custom programs as well as the OntoFox^2^ web tool [13]. The OntoFox tool implements the Minimum Information to Reference an External Ontology Term (MIREOT) principle [14]. MIREOT is a practice by which one imports a selected set of terms from another ontology rather than including the whole ontology, as importing in OWL would do. Where relevant terms were not present in an existing ontology, we created new terms. Each new term was assigned an Internationalized Resource Identifier (IRI) [15], a human-readable label, a definition or documentation, and the name of the term’s editor(s). When appropriate, other metadata was included, such as the reference source for a definition and comments about a term’s definition such as its rationale, scope, and usage. Throughout the ontology development process, the definitions were reviewed multiple times by team members. In the following sections, we discuss the methods for acquiring the necessary terms.

### Ontology architecture

The OHD is constructed in line with a number of OBO Foundry principles. The OBO Foundry [16] is a collective of ontology developers who are committed to collaboration and adherence to shared principles. The mission of the OBO Foundry is to develop a family of interoperable ontologies that are both logically well-formed and scientifically accurate. OBO Foundry principles include use of the Basic Formal Ontology (BFO) [17], an upper-level ontology, use of a standard IRI identifier space, reuse, where possible, of other Foundry ontologies, and the inclusion of a textual and, where feasible, logical definition for each class and relation.

#### Ontology reuse

The OHD uses BFO as its upper-level ontology. BFO is designed as a domain-independent ontology based on principles of ontological realism [18] As an upper-level ontology, BFO establishes categories such as material entities, processes, time, space, and realizable entities (properties), as well as relations among them, such as the relation between a participant and a process they participate in.

We reuse a number of classes and relations from existing OBO Foundry ontologies, such as the Foundational Model of Anatomy (FMA) [19] and the Ontology for Biomedical Investigations (OBI) [20].

This construction methodology serves two purposes. First, it allows us to leverage the experience of the developers of OBO ontologies. Second, adhering to OBO standards and precedents makes the OHD more easily interoperable with other OBO ontologies [16], and this allows developers to reuse our classes and provide feedback on how to improve the OHD. A summary of the reused ontologies is provided in Table 1.

**Table 1:** Summary of ontology reuse in OHD

Ontology	Classes/relations reused or specialized
Basic Formal Ontology (BFO)	upper-level ontology used to coordinate other OBO ontologies
Ontology for General Medical Science (OGMS) [21]	health care entities; e.g., patient role, visit, disorder
Foundational Model of Anatomy (FMA)	anatomical entities; e.g., jaw, tooth, tooth surface
Ontology for Biomedical Investigations (OBI)	relations between processes to entities; e.g., restoration procedure has specified input some tooth
Information Artifact Ontology (IAO) [22]	information entities in the dental health care domain; e.g., billing codes, goals of dental procedures
Ontology of Medically Related Social Entities (OMRSE) [23]	gender of patient
Common Anatomy Reference Ontology (CARO) [24]	male and female organism

Classes from the ontologies listed in Table 1 are then extended to encompass entities in the oral health domain. At present, this includes classes for representing teeth and tooth surfaces, dental procedures, patients, providers, restoration materials, dental findings, and billing codes. Each of these classes is discussed in the following sections.

#### Anatomical structures

We use the FMA’s classes to represent anatomical structures, such as jaws, teeth, and tooth roots. However, in our initial construction of the OHD, we found that the FMA was not adequate for representing surfaces of teeth. The FMA’s class *surface of tooth* is used to represent the two-dimensional curved plane that forms the outer boundary of a tooth. This is not suitable for representing the portions of enamel into which restoration material is placed. Thus, we added the class *surface enamel of tooth* to represent the portions of enamel that constitute a tooth’s anatomical crown. The need for this class was reported to the FMA’s curators, and the FMA now includes the class *surface layer of tooth*^3^ to address this.

Until recently, the FMA was authored in a representation system called Protégé Frames. In order to use it within the OBO framework we needed to translate from the native frames version to a version that integrates with OBO ontologies. As part of that translation, classes in FMA were placed as children of the appropriate BFO or OBO classes. Second, we needed to translate the frames expressions [25] to OWL before we could use it with the other classes OBO classes.

#### Patients and health care providers

A given dental procedure (such as an oral evaluation or restoration procedure) will minimally involve a patient and the dental health care provider. We define for the patient and health care provider roles that characterize the way in which patients and providers participate in dental procedures.

In BFO, roles are realizable entities which are in turn dependent entities. A dependent entity is one that cannot exist unless the entity bearing the role exists. For example, a particular patient role cannot exist unless the organism that bears the role (i.e., the patient) exists. A role is optional in the sense that an entity may gain or lose a role without its physical makeup being changed. For instance, a person may cease to be patient at some practice due to the practice going out of business. The practice’s going out of business is an event that is external to the person, and, thus, does not necessitate that the person is somehow physically changed. Roles are realizable in the sense that their existence can be manifested in a correlated process. For instance a dental hygienist role is *realized* when the hygienist engages in processes related to their profession, such as plaque removal and application of fluoride treatment. Roles and other dependent continuants *inhere,* or are borne by, material entities.

Employing this distinction between roles and their bearers, we define the types *dental health care provider* and *human dental patient* by first defining the appropriate roles for each kind of entity, and then defining providers and patients as being bearers of the roles^4^:

A *dental health care provider role* is a role that inheres in a person who is licensed to provide dental health care and is realized in a health care process.

A *dental health care provider* is a human being who bears a *dental health care provider role*.

A *patient role* is a role that inheres in a person and is realized by the process of being under the care of a physician or health care provider. (OGMS)

A *dental patient role* is a *patient role* that is realized by the process of being under the care of a dental health care provider.

A *human dental patient* is a human being who bears a *dental patient role.*

In order to define the patient’s gender, we use the gender role types from the Ontology of Medically Related Social Entities (OMRSE). The OMRSE is a realist representation of medically related social entities developed to cover demographics data and common roles of people in healthcare encounters for reuse in the context of the OBO Foundry. The gender role types are defined as follows:

A *gender role* is a human social role borne by a human being that is realized in behavior which is considered socially appropriate for individuals of a specific sex in the context of a specific culture. (OMRSE)

A *female gender role* is a *gender role* borne by a human being that is realized in behavior which is considered socially appropriate for individuals of the female sex in the context of the culture in question. (OMRSE)

A *male gender role* is a *gender role* borne by a human being that is realized in behavior which is considered socially appropriate for individuals of the male sex in the context of the culture in question. (OMRSE)

Female and male dental patients are then simply defined by relating the patient to the female and male gender roles:

A *female dental patient* is a *human dental patient* who bears a *female gender role*.

A *male dental patient* is a *human dental patient* who bears a *male gender role*.

Using roles to define patients and dental health care providers has two advantages. First, because roles are formally defined, they represent the semantics for how an entity participates in a procedure. That is, for a given dental procedure, the patient participant is the entity whose participation realizes the *dental patient role*, and the provider participant is the entity whose participation realizes the *dental health care provider role*. In contrast, field names and values in relational databases are purely syntactic.

Second, by using gender roles instead of anatomical sex to represent male and female dental patients, we allow for the possibility that the gender a patient assigns to himself or herself may differ from the patient’s anatomical sex (at birth), matching the common practice of recording patient-reported gender in clinical systems. In those cases in which biological sex needs to be represented, the OHD includes CARO’s types *female organism* and *male organism*:

A *female organism* is a gonochoristic organism that can produce female gametes. (CARO)

A *male organism* is a gonochoristic organism that can produce male gametes. (CARO)

Using these classes, a patient’s biological sex can then be defined according to biological criteria rather than gender selection.

#### Dental procedures

We define the class *dental procedure* as a subclass of OGMS’ *health care encounter class*:

A *health care encounter* is a temporally-connected health care process that has as participants an organization or person realizing the health care provider role and a person realizing the patient role. The health care provider role and patient role are realized during the health care encounter. (OGMS)

A *dental procedure* is a *health care encounter* that realizes a *dental patient role* in which the patient undergoes a diagnostic or therapeutic process.

**Fig. 1:**
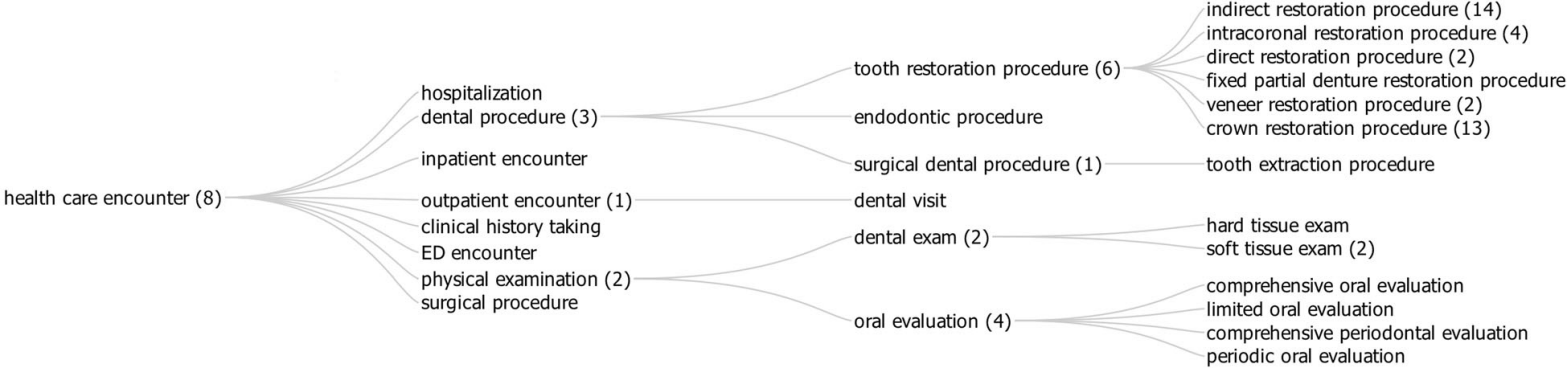
A portion of the hierarchy of health care encounters in OHD. Numbers represent the number of direct subclasses for a class, some not shown for reasons of space

As illustrated in Fig. 1, specific dental procedures are then defined by specializing the *dental procedure* class. For instance, *endodontic procedure*, *surgical dental procedure*, and *tooth restoration procedure* are defined as follows:

An *endodontic procedure* is a *dental procedure* that is performed on the pulp chamber and/or root canal of a tooth, or a part thereof.

A *surgical dental procedure* is a *dental procedure* in which there is structural alteration of soft tissue or bone in or around the oral cavity by incision or destruction of tissues or by manipulation with instruments causing localized alteration or transportation of tissue, including lasers, ultrasound, ionizing radiation, scalpels, probes, and needles*.*

A *tooth restoration procedure* is *dental procedure* in which either a whole tooth or a part of a tooth is replaced by dental restoration material in order to reestablish the tooth's anatomical and functional form and function.

More specific surgical and restoration procedures are then defined as subclasses of these terms. For example, a non-exhaustive set of surgical and restorative procedures defined in the OHD include:

A *tooth extraction procedure* is a *surgical dental procedure* that removes a tooth from the oral cavity.

A *crown restoration procedure* is a *tooth restoration procedure* whereby an artificial crown replaces all or part of the natural dental crown.

A *direct restoration procedure* is a *tooth restoration procedure* in which the dental restoration material is placed in the tooth via some direct dental material insertion process.

An *indirect restoration procedure* is a *tooth restoration procedure* in which the dental restoration material is placed in the tooth via some dental material tooth attachment process.

An *intracoronal restoration procedure* is a *tooth restoration procedure* in which a dental restoration material is placed into a site that is located in the crown of the tooth.

A *veneer restoration procedure* is a *tooth restoration procedure* in which a thin layer of material (i.e., a veneer) is placed over one or more surfaces of the tooth for purposes such as improving the aesthetics of the tooth or protecting the tooth's surface from damage.

Patients and providers are related to dental procedures using BFO’s *has participant* and *realizes* relations. The *has participant* relation is a general way of relating processes to the entities involved in them. For example, an oral evaluation (minimally) has as participants the patient undergoing the evaluation and the provider doing the evaluation. The *realizes* relation holds between a process and a realizable entity such as a role. A role is defined in terms of what bears it, what process realizes it, and the manner in which the bearer participates in the process. As an example, consider the aforementioned *dental patient role* and *dental health care provider role*. When a dental procedure is performed, the procedure *realizes* the roles of the patient and provider. The person upon whom the procedure is performed acts (or behaves) as the dental patient and the person doing the procedure acts (or behaves) as the provider. In this way, the dental procedure *realizes* the *dental patient role* of the patient and the *dental health care provider role* of the provider.

BFO defines temporal regions and a relation *occupies temporal region* that defines the temporal span of a process. However, we don’t use this representation for two reasons. First, there isn’t yet an established OBO practice for specifying concrete dates. Second, the time of a procedure is recorded only to the granularity of a day. Pending development of representations that accommodate these issues, we defined a date property, *occurrence date*, that relates a process to an xsd:dateTime some time during which the process occurred. Another data property *birth date* was defined to relate a patient to their date of birth.

To characterize the way in which a tooth participates in a specific dental procedure, we define roles that are borne by the tooth and realized in the appropriate corresponding procedure. For example, in order to represent that a tooth undergoes a root canal treatment, we specify that a tooth bears a particular *tooth to undergo endodontic procedure role* and this role is then realized in a particular *endodontic procedure.*

For procedures that involve restorative materials, we define a *dental restoration material role* that is borne by (i.e. possessed by) the restoration material. The role helps define the material in a domain-neutral way. All gold is metal, but not all gold is used in dental restorations, just those that bear the dental restoration material role.

This role is then realized by the corresponding restoration procedure. For instance, an *intracoronal restoration procedure* (see above) realizes the *dental restoration material role* of the material that is placed inside the crown of the tooth. In procedures that involve a specific kind of material, we use OBI’s *has_specified_input* relation to express that a procedure uses that material. For example, an *amalgam filling restoration* is defined as follows:

An *amalgam filling restoration* is an *intracoronal restoration procedure* that uses amalgam to restore the tooth.

As part of the logical framework of the OHD, we then include the axiom that an *amalgam filling restoration has_specified_input* some portion of *amalgam restoration material*.

#### Restoration materials, restored teeth, and prosthetics

For dental procedures that involve the use of restoration materials (e.g., amalgam), we define the restoration materials in terms of the role the material has in replacing portions of the tooth. In general, dental restoration material has the role of serving as a prosthetic, that is, the material has the role of replacing a missing body part. However, not all prosthetics replace the function of the missing body part, for example, a prosthetic eye cannot see, although it still functions to maintain the shape of the skull near the eyes. To address this, we define the term *functional prosthetic role* to represent a prosthetic that performs the function of the replaced body part. Since dental restoration materials perform the function of parts of the tooth they replace, we define *dental restoration material role* as a subtype of *functional prosthetic role*:

A *functional prosthetic role* is a *prosthetic role* that is realized by activities in which the material entity (bearing the role) is used a manner that is similar to how the body part that the prosthesis replaces would be used.

A *dental restoration material role* is a *functional prosthetic role* that is borne by a portion of dental restoration material and is realized in a tooth restoration procedure in which the restoration material becomes part of a restored tooth.

*Functional prosthetic role* is not a term that is specific to oral health and so should lie outside the scope of the OHD. Our inclusion of it demonstrates a necessarily pragmatic approach in which we sometimes define general terms necessary to capture a term within our domain when they are not yet present in a more appropriate ontology. In this case, the term would more properly belong to OGMS.

Outright replacements of teeth such as pontics or implants also have a *functional prosthetic role*. We do not consider, however, restored teeth to be, as a whole, prosthetic. We introduce the term *functional tooth,* as the union of original teeth, *restored teeth*, and *prosthetic teeth.*

The restoration materials themselves are defined as a kind of *processed material* that bears a *dental restoration material role*:

A *processed material* is a *material entity* that is created or changed during material processing. (OBI)

A *dental restoration material* is a *processed material* that bears a *dental restoration material role*.

Specific restoration materials are defined according to the substance that constitutes them or how they are used. For instance, *amalgam dental restoration material* and *resin dental restoration material* are defined as follows:

A *metal dental restoration material* is a *dental restoration material* that consists mostly of metal atoms.

An *amalgam dental restoration material* is a *metal restoration material* that consists of a silver-colored, metallic alloy which is composed of a mixture of mercury and other metals.

A *resin dental restoration material* is a tooth-colored *dental restoration material* made from a mixture of resin, silica and other materials used in direct restorations.

By using the term *dental restoration material role* to define *dental restoration material*, we are then able to relate the restoration materials to the tooth restoration process that use these materials by specifying that the procedure *realizes* the *dental restoration material role* borne by the *dental restoration material*.

Finally, we relate a particular instance of a restoration material to the anatomical part it restores using the *is dental restoration of* relation. For example, an amalgam filling procedure that restores the occlusal surface of the right upper first secondary molar (i.e., ADA tooth number ‘3’), is represented as an instance of *amalgam dental restoration material* that *is dental restoration of* the particular *occlusal surface* which *is part of* the patient’s *tooth 3*. We use a relation here because of an unusual temporal arrangement: the restoration material and the tooth surface don’t necessarily exist at the same time.

#### Dental findings

In the OHD, we make a clear distinction between information that is part of the patient’s medical record, and the oral condition or treatment referred to by the information. To represent information, we define the class *dental finding* as a specialization of OGMS’s *clinical finding* class:

A *clinical finding* is a representation that is either the output of a clinical history taking or a physical examination or an image finding, or some combination thereof. (OGMS)

A *dental finding* is a *clinical finding* that is a specified output of a dental exam and is about the oral cavity, maxillofacial area, and/or the adjacent and associated structures, or their parts, or pathological anatomical entities derived from them.

We are then able to further specialize dental findings as needed. For instance, a *caries finding* that a patient has caries on some tooth is defined:

A *caries finding* is a *dental finding* that indicates a carious lesion of a tooth.

Findings are related to their targets using IAO’s *is about* relation. For example, a particular *caries finding* has as its target a particular tooth in a patient’s mouth. This is represented as instance of a *caries finding* that *is about* the patient’s particular *tooth*.

For representing findings about missing teeth, we were faced with the issue that, following the principles of ontological realism, one cannot have a finding about a non-existent entity. For instance, if a patient is missing his right upper first secondary molar (i.e., tooth 5), you cannot have finding about this tooth because there is no tooth that is the target of the finding. To address this, we defined a *missing tooth finding* to be about a patient’s mouth, and we made use of the strategy put forth in Hoehndorf et al. (2010) to represent anatomical entities that lack a particular part [26]. In brief, Hoehndorf et al. represent such entities by negating the *part of* relation that holds between an entity and one of its parts. A mature red blood cell, for example, does not have a nucleolus. Thus, a red blood cell is formally defined (i.e., ‘=df’) as follows:

*red blood cell* =df *blood cell* and (not *has part* some *nucleus*)

Extending this strategy to missing teeth findings, we formally define a *missing tooth finding* as being about a mouth (i.e., *is about* some *mouth)*, and a missing tooth finding for a particular tooth as being about a dentition that lacks a particular tooth. For instance, a *missing tooth 5 finding* is formally defined as follows:

*missing tooth 5 finding* =df *missing tooth finding* and *is about* some (*secondary dentition* and (not *has part* some *tooth 5*))

In natural language, this formal definition is rendered as:

A *missing tooth 5 finding* is a *missing tooth finding* in which *tooth 5* is found to be missing.

The missing tooth findings for the other teeth are then defined using the same approach as a *missing tooth 5 finding*, but with ‘*tooth 5*’ being replaced by the relevant tooth (e.g., *missing tooth 6 finding*, *missing tooth 7 finding*, etc.).

#### Billing codes

Given the ubiquity of current dental terminology (CDT) codes in electronic dental record systems, we need to represent these codes in the OHD. Since CDT codes are a kind of information, we defined them as a subclass of IAO’s *centrally registered identifier* (*CRID*). A *CRID* is a symbol that is registered as part of some organization. For example, a social security number is a *CRID* this is registered with the Social Security Administration. Similarly, CDT codes are registered and developed by the American Dental Association [27], and thus, the *current dental terminology code* class is defined as:

A *current dental terminology code* is a centrally registered identifier that is maintained by the American Dental Association and used for recording dental services provided. It is typically used on the patient record, and when reporting procedures on a paper or electronic submission.

To date, the OHD contains only a subset of total number of CDT codes, and most of the represented codes lack definitions. This is because *current dental terminology code* subclasses were created by using computer programs to extract information about CDT codes from the relevant ADA documentation [28].

### Translating dental EHR data into OHD-based representations

In parallel with developing the OHD’s class hierarchy, we developed methods to programmatically translate dental EHR data into OWL statements. For this, we wrote custom Common Lisp^5^ programs to extract and translate dental EHR data (data source described below) stored using Eaglesoft Dental Practice Management and Imaging Software.^6^ This process consisted of three steps.

First, we extracted data from the dental EHR system using SQL queries issued by our Common Lisp program. In order to facilitate access to the EHR data, we combined several of the Eaglesoft tables into a single table named ‘patient_history’. This made queries simpler to write and understand. That is, instead of having to do a number of joins across multiple database tables, we were now able to query just one table. For example, the following query extracts data about patients who had undergone single surface amalgam restorations:

SELECT * FROM patient_history WHERE ada_code LIKE 'D2140'

Second, once extracted, the dental EHR data was translated into OWL statements about instances of the entities involved using the Lisp Semantic Web Library (LSW) [29], a Common Lisp library whose syntax is similar to OWL’s functional syntax [3]. For example, the following OWL functional syntax statements declare that a particular individual is an instance of a tooth:

Declaration (NamedIndividual(obo:tooth_instance))

ClassAssertion (obo:FMA_12516 obo:tooth_instance)

In LSW, these statements are written as follows:

(declaration (named-individual !obo:tooth_instance))

(class-assertion !’Tooth’@ohd !obo:tooth_instance)^7^

The close affinity of the LSW syntax to the OWL functional syntax, as well as its ability to reference classes by name instead of IRI, allows us to write OWL statements that we can easily understand and evaluate. This is in stark contrast to OWL statements represented as RDF/XML, which are not easily understood by humans. The output of the Common Lisp program is a number of OWL files that together contain an OWL representation of the dental EHR data.

Third, the OWL files are loaded into a semantic database, or triple store, that uses the OHD as the schema for representing the data. This allows for data to be easily queried and analyzed (discussed in the *Results* section). For this project, we used the GraphDB SE version 7.2 triple store,^8^ a semantic database with integrated reasoning that is built on Semantic Web standards. To verify that data was translated correctly, we ascertained that the number of entities in the triple store matched the number in the dental EHR relational database. For example, we queried the number of patients and dental procedures in the triple store and compared those results to SQL queries used to extract the same information from the dental EHR.

## Results

### Summary of the OHD

In its present state, the OHD contains 1947 classes, and 59 relationships. Tables 2 and 3 summarize the source of these classes and relations.

**Table 2:** Summary of number of classes used in the OHD

Ontology	Number of Classes
Foundational Model of Anatomy	1515
Oral Health and Disease Ontology	192
Current Dental Terminology codes	174
Ontology for General Medical Science	74
Basic Formal Ontology	32
Information Artifact Ontology	14
Ontology for Biomedical Investigations	13
Common Anatomy Reference Ontology	3
Ontology of Medically Related Social Entities	3
NCBI Taxon	1
**Total**	**1947**

**Table 3:** Summary of number of relations used in the OHD

Ontology	Number of Relations
Basic Formal Ontology	38
Foundational Model of Anatomy	12
Ontology for Biomedical Investigations	5
Oral Health and Disease Ontology	3
Information Artifact Ontology	1
Total	59

In the above tables, it is important to point out the relatively low number of new classes and relations created specifically for the OHD. That is, 192 out of 1947 classes and 3 out of 59 relations were specifically developed for the OHD. This high amount of reuse demonstrates the effectiveness of the OBO Foundry principle of having distinct scientific communities develop rigorous interoperable ontologies. Without OBO Foundry ontologies that follow these principles, we would have to draw upon other terminologies, such as SNOMED CT,^9^ that don’t provide the same sound basis, clarity, level of documentation, or instance-orientation that characterizes the OHD.

### Translation of practice data

The primary data source for this analysis was the relational database from a dental EHR containing de-identified dental records for 7337 patients from a single dental practice spanning the years 1999–2011. Of these patients, approximately 4500 had treatment records. The practice used Eaglesoft (Patterson Dental, Effingham, IL), a leading dental EHR system in the U.S. In total, the database contained 232,270 records that pertained to patients’ dental health history.

After receiving IRB approval, we used the methods described in *Section 3.3* to translate the dental EHR data into OWL. Where there were questions regarding what certain data meant, we consulted the vendor of the system and the practice clinician.

The following kinds of procedures were translated: fillings, crowns, onlays, inlays, veneers, endodontic procedures, surgical extractions, and oral evaluations. Table 4 summarizes the resulting number of translated procedures:

**Table 4:** Summary of the number of procedures translated in the OHD

Procedure	Total
Fillings	22,252
Crowns	12,636
Onlays	1269
Inlays	365
Veneers	877
Endodontic procedures	1441
Tooth extractions	999
Oral evaluations	28,566
Total	68,405

The SPARQL query used to retrieve the number of translated procedures is provided in Additional file 1. The translated data were then loaded into a GraphDB SE (version 7.2) triple store using GraphDB’s OWL2-RL automated reasoner. Result sets for analysis were obtained by querying the triple store using SPARQL 1.1.

### A clinical use case: longevity of restorations

As a proof of concept for using the OHD structured data to analyze dental EHR data we performed an analysis of the longevity of tooth restorations. For our study the steps involved were

1. define what would be considered a restoration failure;
2. define a set of correlates for the analysis: We look at patient, gender, age, tooth position and number of surfaces restored;
3. design the queries to extract the relevant information;
4. prepare the database for the sorts of queries we would be making; and
5. using the R statistics program to run the statistics and plot curves.

#### Defining restoration failure

There appears to not be a standard approach to studying restoration longevity. Prior studies have used a variety of criteria to define or categorize failure, and different correlates. We describe next a representative set.

Bogacki et al. [30] defined failure as replacement of a restoration on the same surface. They censored cases where a larger restoration replaced the initial one (e.g., a one-surface restoration replaced by a two-surface restoration) or at the last known patient visit. Correlates included restoration type, prior history, provider and patient age, tooth location, year of treatment, and whether the provider changed.

Gulambi et al. and Redman et al. [31, 32] categorized failures into major and minor based on United States Public Health Services USPHS score, and analyzed major failures and total failures separately. Major failures were defined as restorations that required complete replacement. Restorations were censored if there was no other event at last follow-up. Correlates included patient gender, provider, etiology of tooth wear, material, opposing dentition and incisal relationship.

In Janus et al. [33] failure was defined as the tooth being lost due to extraction, or the tooth requiring an additional restoration, crown, or other treatment such as an endodontic treatment. Only a single tooth from any patient was chosen to minimize dependence. Restorations without further activity were censored as of last date the patient was seen. Correlates were gender, race, age, site, type of restoration, and whether the initial restoration was supervised by a specialist. We defined failure similar to Janus et al. A restoration was considered a failure if there was a subsequent restoration on any of the surfaces that were initially restored. Cases were censored if there was no failure the last time the patient was seen. If there was no encounter after the restoration, we did include the restoration in our analysis.

#### Correlates

Correlates were chosen for relevance and based on their availability in our data set. We considered the patient’s age at restoration, gender, whether the tooth was anterior or posterior, and how many surfaces were initially restored. Age was broken into two groups: below and above 40. When an initial analysis did not find significance using the exact number of surfaces restored, we chose to group the data into two categories – those where a single surface was restored and those where more than one surface was restored.

Etiology and condition at time of failure are likely to be recorded in progress notes, but could not be easily extracted from our source data. While provider information was ostensibly recorded, we were told it was not reliable.

#### Database preparation

After translation, we noted an issue. We had given dental visit an occurrence date, but not the processes that occurred during visit, such as the exams or restorations. Ideally we would add an OWL property chain has_part o occurrence_date - > occurrence_date, however OWL2 only has object property chains. Instead we used a SPARQL Insert command to update this as shown in Additional file 1.

**Fig. 2:**
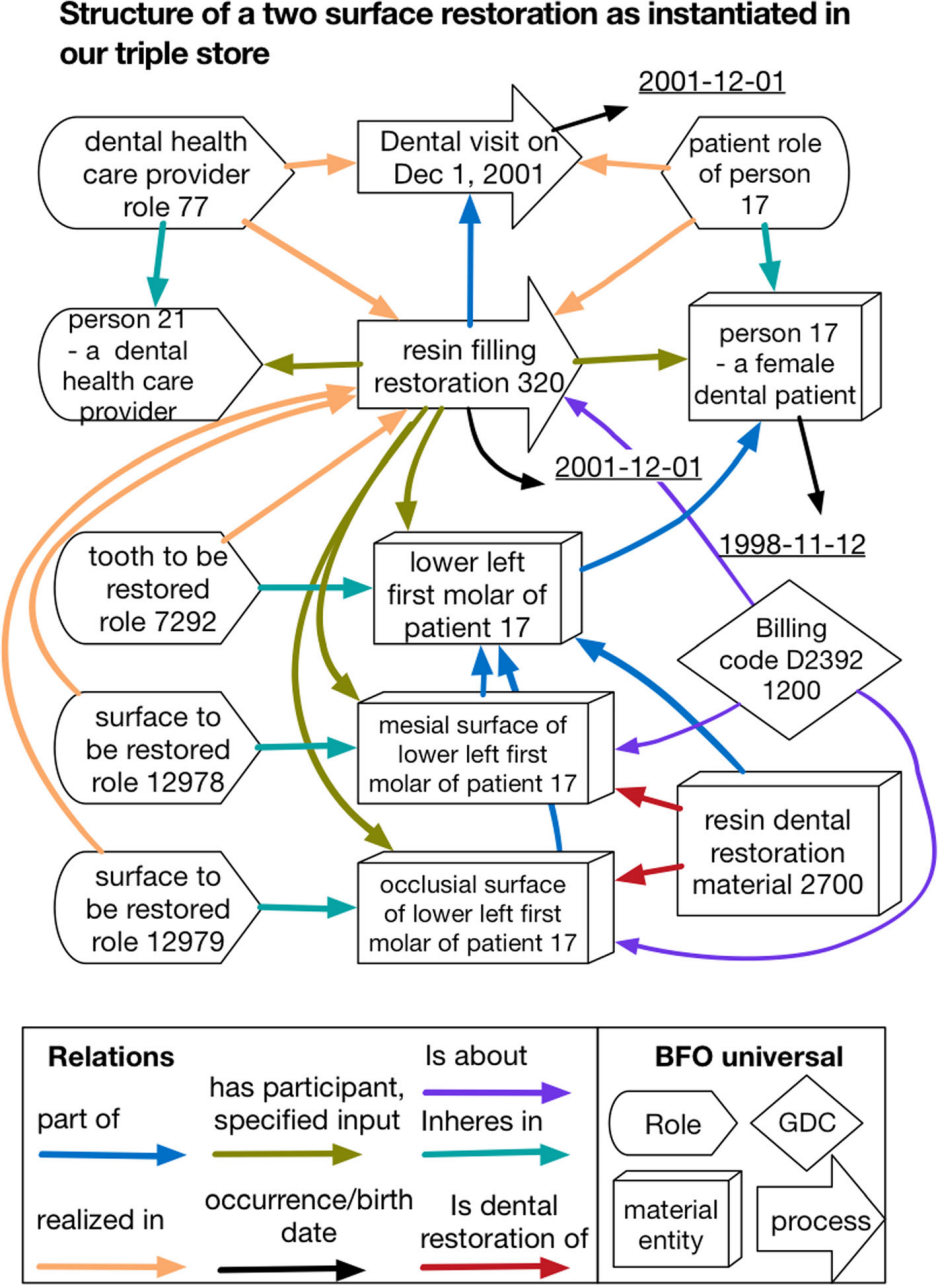
Illustration showing instances used in representation of a two-surface resin restoration. Each box is an OWL individual. Arrows indicate the relations among the individuals, and box shape indicates the upper level BFO universal which it instantiates. In some cases, a proximate superclass is listed after a dash. In other cases, the label until the instance number or ‘of’ names the proximate class. Where ‘of’ is used it indicates a functional relation. Underlined dates are data values

To make queries both efficient and clear we created a relation that linked, in chronological order, each patient encounter. For each patient, *next_encounter* relates each encounter to the following encounter with the patient. Note that since encounters may be part of other encounters, and that our time granularity is a day, there may be several *next encounters* after a single one. To create the chain of encounters, we used SPARQL’s ability to test whether a variable is bound in a solution, using the strategy shown in Fig. 2. We did a query for triples of events in order, but we filtered any solutions in which the middle encounter was bound.

*subsequent_encounters* is asserted as a transitive superproperty of next_encounter, making relatively easy to construct queries looking for ordered pairs of type of processes.

#### Queries

The general schema for the SPARQL queries for pairs of events for survival analysis was:

**Table:** 

?first_event rdf:type <type of restoration>:. *<bind tooth and surface>* ?first_event subsequent_encounter: ?later_event. *<constrain second event>* ?later_event occurrence_date ?date. *<bind survival analysis correlates>* Filter for minimal ?date of ?later_event	

Table 5 lists the kinds of failures and conditions that were used as second events. A query retrieving restorations whose second event is one of the four conditions is captured in the query in Additional file 1.

**Table 5:** Failure event type and constraint

Type of second event	Second event constraint
Restoration or inlay	Same tooth and surface
Endodontic procedure	Same tooth
Crown replacement	Same tooth
Extraction	Same tooth
Last recorded encounter	No later encounter

All queries returned information solely about secondary teeth. Out first query retrieved the date of the restoration as well as the date of the last recorded encounter with the patient, the latter used as right-censored events in the survival analysis when there was no recorded failure. In that case the constraint on the later event is that there is no subsequent event, i.e. there is no subsequent_encounter relation. When a restoration was placed during the last patient visit on record, it was not included in the analysis.

The second query looked for pairs of restorations and failures. The target restoration type here was *resin filling restoration*, and we considered as failure either a restoration or inlay on any surface of the first restoration, or replacement by a crown, or an extraction of the tooth, or an endodontic procedure on the tooth. It should be noted that we did not need to use an explicit list of dental codes for our query, as is commonly the case. Instead the dental procedure hierarchy provides us the ability to query all instances of, e.g., endodontic procedures and its subclasses. Should they be necessary, queries can still be made in terms of CDT codes since axioms relate CDT codes to what they are about.

#### Statistical analysis

Our dataset included 13,922 resin restorations, of which 12,704 had follow up. Table 6 provides a breakdown of the events by correlate, giving, for each group the number of patients, the number of events (failures + censored) and the number of failures.

**Table 6:** Breakdown by correlate of events for survival analysis

Correlate	No. Patients	No. Events	No. Failures
Gender female	1441	7114	1358
Gender male	1117	5590	1251
Anterior tooth	1195	3692	989
Posterior Tooth	2234	9012	1620
Age < 40	1082	5159	824
Age over 40	1545	7545	1785
Single surface	2016	6216	1346
Multiple surface	2021	6488	1263

For analysis we used the R packages survival [34], simPH [35], and ggsurv from GGally [36] for the Kaplan-Meier plots. The muhaz [37] package was used to compute the hazard function. With a cutoff of .05 for *p*-value all correlates, all were statistically significant. Table 7 shows *p* value and relative hazard for significant correlates.

**Table 7:** Significant correlates significance and effect size

Correlate	p	Hazard difference
posterior vs anterior	<.001	23% decrease
Male vs female	<.001	16% increase
Age 40+ vs < 40	<.001	40% increase
multiple vs single surface	0.0019	12% decrease

**Fig. 3:**
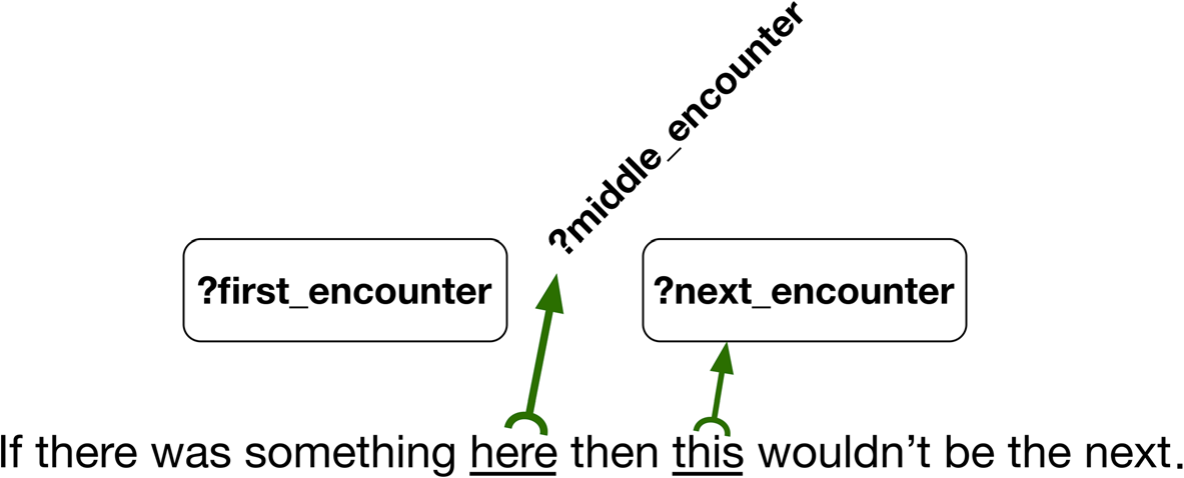
Construction of the next encounter relation

Figure 3 shows Kaplan-Meier curves for overall longevity and comparison curves for each correlate. Grayed area are 95% confidence intervals. Median survival, overall, is somewhere in the range of 12 years, falling to 10.5 years median survival for those older than 40. Our results on longevity most resemble Bogackiet et al. [30]. They don’t seem to concord with Redman et al. who found median survival to be substantially less at under 5 years [31].

The hazard rate was plotted once it was determined that there was a surfeit of early failures. The plot clearly shows this with elevated hazard in the first 2 years. Code to perform the analysis is available at https://github.com/oral-health-and-disease-ontologies/ohd-ontology/blob/master/src/analysis/survival.r.

## Discussion

We have outlined the principles used to create the OHD classes and relations, and discussed how data translated into OWL can be queried and analyzed. While there have been recent efforts to use instance data in a clinical setting [38], this work represents the first example of end-to-end use of BFO and OBO-ontology structured instance data for clinical research in healthcare. By using practice data and demonstrating how to answer a clinical research question, we have demonstrated that the approach is practical with current technology.

Along the way to implementation we were faced with a number of situations where existing ontologies alone were inadequate or where choices of how to use them were not obvious. We chose to represent CDT codes as information artifacts beside, so to speak, representations of the processes those codes refer to, to make room for, but not tie us to, a single coding system.

The treatment of time in current OBO OWL ontologies is inadequate [39]. In different cases we variously ignored it, addressed it with an alternate ontology pattern, or used a workaround. We use the *is part of* relation without a time index despite BFO’s part relation being time-dependent. A common error in representing processes is to define them in terms of participants *having* certain roles. The *has role* relation should also be time-indexed and, importantly, entities with roles can and do participate in processes where those roles are not relevant. We use the role-realization pattern to clearly relate roles, when they are relevant, to a process and this has an added benefit of having correct temporal scope without specifying a time index. Our use of *occurrence date* is a workaround for the lack of a way of specifying concrete times and also makes a choice about temporal granularity, something else not clearly addressed in current OBO.

We needed to work with natural, restored and prosthetic teeth. Anatomy ontologies are typically canonical, and so do not address these cases. There have been various efforts to address this, for example in understanding how non-canonical phenotypes relate to anatomy [40]. Here, we introduced a representation of prosthetics and introduced the term functional tooth, to capture the necessary distinctions. Some of the terms are not specific to our domain and we have proposed they be added to OGMS.

It was not clear at the start how to have performant queries that retrieved pairs of related encounters such as one in which a restoration is done and a later one requiring a root canal because the first failed. Creating the *next_encounter* and *subsequent_encounter* relations and using them for such queries proved to be effective, both computationally and cognitively.

Ontologies sometimes did not have terms in their domain that we needed. While the FMA is incredibly detailed, we discovered it did not give us a representation of the substantial (as opposed to two-dimensional) surface of teeth. We created the necessary terms and worked with the developers of the FMA to have them added, a success for the collaborative approach espoused by the OBO Foundry.

We see five benefits of the approach we used. The first, which can be easily overlooked, is that building the OHD aided in understanding the domain and data by forcing us to use unambiguous terms. As an example, consider the term ‘restoration’. Depending on the context, a practitioner may mean the process of restoring a tooth or the material used to restore a tooth. These two uses of ‘restoration’ are interrelated: The process of restoring a tooth requires the use of restoration material. However, the process and the material are distinct types of entities.

The second benefit is that we are not reliant upon a particular coding system, but can still use one or more effectively. For instance, in the United States, teeth are denoted according to the Universal Tooth Numbering System [41], which uses the numbers 1–32 for permanent teeth and the letters A-T for primary teeth. But, other countries use the World Health Organization (WHO) notation system, which denotes teeth using combination of numbers to represent the quadrant of the mouth and the tooth’s position in the quadrant. The OHD, in contrast, represents teeth using anatomical classes from the FMA. For example, the OHD represents a patient’s upper right wisdom tooth using the FMA class Right upper third secondary molar tooth, which is also denoted as ‘1’ in the Universal Tooth Numbering System and ‘18’ in the WHO system. Thus, different tooth coding systems can be mapped to the same FMA class. Similarly, whereas dental representations often use CDT codes, which also are used for billing, the OHD represents types of procedures and codes separately. When multiple codes are meant to refer to the same thing, their meeting point can be, for example, a single process type.

The third benefit of our approach is that using the OHD allows for queries that leverage the logical structure of the ontology. Two examples of this are hierarchical queries and relational queries.

By design, an ontology behaves such that if you query for instances of a class, instances which are only asserted to be of a subclass (or child class) are queried for as well. That is because they are inferred to also be the parent type. For example, consider a query to retrieve all crown procedures. Using the OHD, this query is straightforward^10^:

**Table:** 

select (count(distinct ?procedure) as ?total_crowns) where { ?procedure rdf:type crown_procedure: }	

However, to do this in a relational database using ADA billing codes alone, you have to account for at least 40 billing codes.^11^

In addition to hierarchical queries, the OHD permits queries that make effective use of the relations between entities. For example, in the OHD, we specify that a tooth restoration procedure must include restoration material during the process. This permits us to query for all procedures that use a specific restoration material instead of having to recall which materials are denoted by ADA codes. The query below leverages the OHD specification to find the number of restoration procedures using resin broken down by the type of procedure.^12^ The result of this query is summarized in Table 8.

**Table 8:** Summary of the number of restoration procedures using resin

Procedure Name	Total
resin filling restoration	13,860
resin laminate veneer restoration	135
resin inlay restoration	13
resin onlay restoration	11
resin with predominantly base metal crown restoration procedure	2
resin crown restoration procedure	1
stainless steel with resin window crown restoration procedure	1

**Table:** 

select ?procedure_name (count(?procedure_type) as ?total) where { ?material_instance rdf:type resin: . ?procedure_type rdfs:subClassOf dental_procedure: . ?procedure asserted_type: ?procedure_type . ?procedure has_participant: ?material_instance . ?procedure_type rdfs:label ?procedure_name . } group by ?procedure_name order by desc(?total)	

**Fig. 4:**
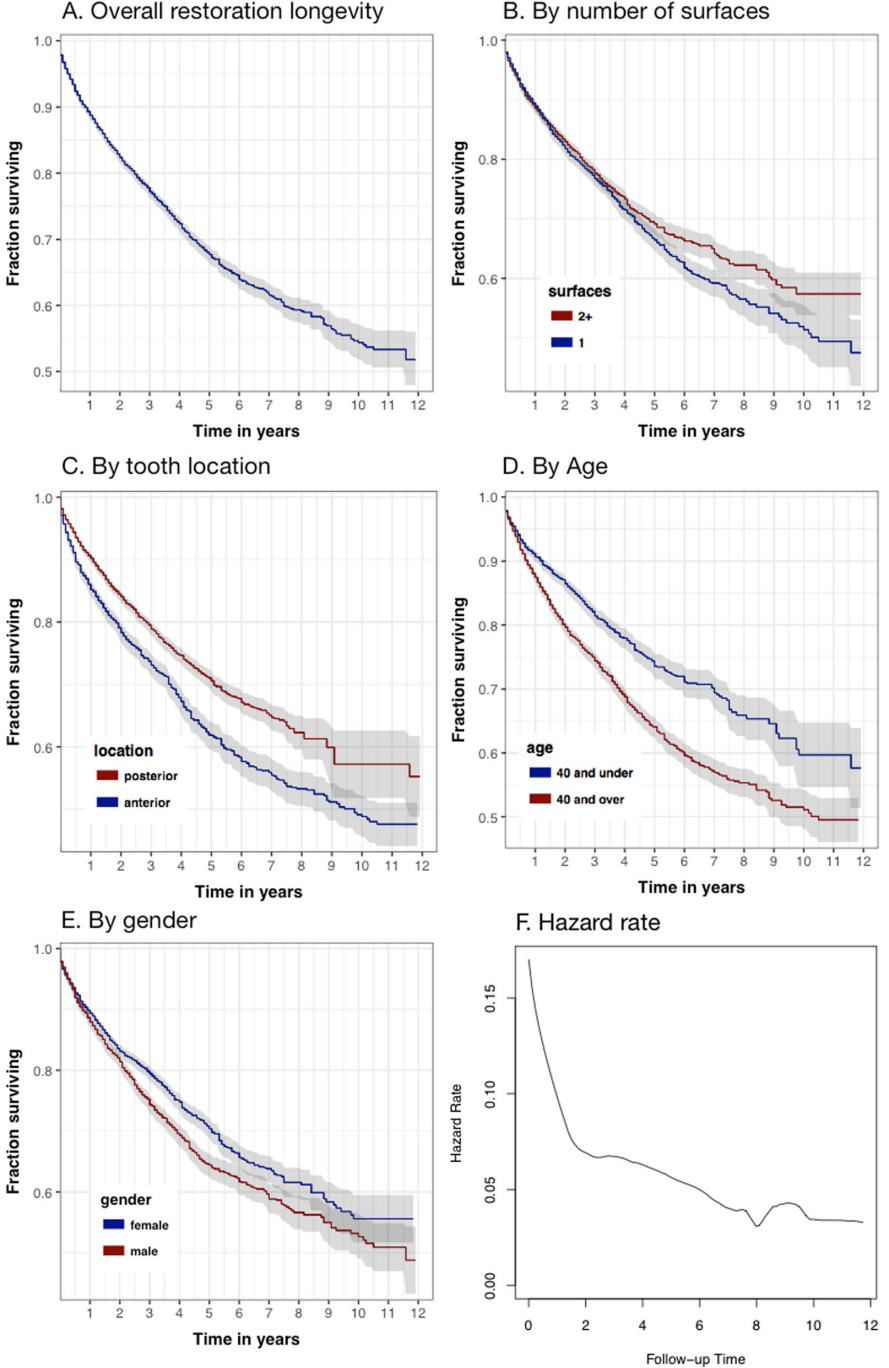
**a-e**: Survival plots **f**: Hazard rate

The fourth benefit is easy generalization to wider domains of medicine and clinical research, and is conferred by our extensive use of instances and types. Consider the representation in Fig. 4. This representation contrasts with typical representations from relational databases in that it more explicitly tracks what happens and include type information making it easier to discover and understand contents. For example, the situation represented in Fig. 4 might be represented in a single row in a relational database.

**Table:** 

**Primary key**	**Provider id**	**Patient id**	**Tooth no.**	**Surfaces**	**CDT Code**
XXXXX	21	17	19	MO	D2392

The relational representation provides economy of space and may be suitable for a self-contained application. However, this representation makes it difficult when it comes to integration in either a data warehouse or a system which records information that goes beyond dentistry. Information needed to interpret it, if even available, typically lies outside the representation. Professionals charged with integrating or querying such data are typically not domain experts. For example, a person who needs to understand the representation needs to understand that each letter in the surface column represents one surface and find a reference for the lettering system. In addition, the representation provides little guidance about how one might go about representing the information in a way compatible with more general medical information about a patient. For example, the provider id implicitly identifies a dentist. In a more general setting, there are a wide variety of types of providers and this information must be made explicit.

By comparison, the representation in Fig. 4 might appear at first glance to be overkill. However, it has two important qualities. First, it is easier to generalize to general medicine – the same schema can easily accommodate procedures of any sort, by providers of any sorts, on parts of the anatomy of any sort. Second, because it creates instances for the entities it removes ambiguities that might hinder careful clinical analyses, a problem identified and addressed in the strategy of *referent tracking* [42]. With this representation one can easily track the particular material used in a filling even when the filling is redone after failure or refer to the specific usage of this CDT code in context, for example in an audit.

The final benefit of our approach is that it allows us to use automated reasoning to infer information. That is, automated reasoners can use OHD’s axiomatic definitions to infer relations between entities that were not explicitly stated as being related. Two examples of this are the OHD’s use of transitive relations and the functional tooth class. A transitive relation is a relation where if two relationships have a common element that is object of the first relation and subject of the other, then the respective subject and object of those relations are also related. For instance, in mathematics the ‘<’ (less than) operator is transitive. Given that 5 < 6 and 6 < 10, you are licensed to infer that 5 < 10. In the OHD, we define the ‘is part of’ as transitive, and use it relate a tooth’s surface to its tooth and a tooth to a patient. Using these relations between surface and tooth, and between tooth and patient, we can now easily query for patients’ tooth surfaces as follows^13^:

**Table:** 

select ?surface ?patient where { ?patient rdf:type patient: . ?surface rdf:type tooth_surface:; is_part_of: ?patient . }	

In this query, the parthood relation between the tooth surface and patient is inferred, and not explicitly stated in the translated data.

In the OHD, a functional tooth is defined to be either a natural tooth, a restored tooth, or a prosthetic tooth. This allows us to easily query for all restoration procedures performed on a tooth regardless of whether the tooth is natural or an implant. However, when translating the data, we do not explicitly state that a tooth or implant is a functional tooth. Rather, we define the axioms that specify the necessary and sufficient conditions for being a functional tooth and let the automated reasoner do the work of classifying the instances. Our reason for classifying an instance of functional tooth in this manner is that as the number of classes to which an instance can belong grows, the complexity of the ontology increases, which likewise increases the chance for misclassifying an instance. By offloading this task to the automated reasoner, we reduce the chance of introducing human error into the process of classifying instances of functional tooth.

## Conclusions & future work

Our goal of developing the OHD was to leverage semantic web standards to provide a common representation of the dental health care domain that exercises good ontology practices and can be generalized to general medicine. In doing this, we reused classes from OBO Foundry ontologies when possible. The advantage benefit from this was twofold. First, we were able to effectively leverage work done by others within the OBO community. Second, we were then able to improve on the OBO ontologies when we found cases that were not yet represented, for instance the surface enamel of tooth class that was added to the FMA as a result of our work. This has the added benefit of enriching the OBO Foundry community in general.

We only translated data from one practice, and while our purpose was not to design a data integration pipeline, building such a pipeline was necessary in order to have data for analysis. Our translation method can be applied to other practices with different dental EHR systems, and efforts are currently under way to integrate data from 99 geographically dispersed dental practices using the OHD. One notable consideration to our approach is that in order to translate dental EHR records, one must learn the intricacies of the dental EHR database schema. We had the advantage of having an open line of communication with Eaglesoft, the practice’s dental EHR vendor, but researchers wishing to adopt the OHD might not have this advantage. Thus, further work needs to be done on how to communicate the necessary steps needed to query a vendor’s database to those who do not have contact with the vendor and to advocate that new systems use an ontology-based representation.

Our use cases for this project primarily focused the representation of tooth restorations (e.g., fillings and crowns), and dental procedures or conditions that would indicate a tooth restoration had failed. We recognize that there are number of other procedures and conditions that need to be represented. The representation of these other entities is part of our ongoing work, as is the application of the methodology in the wider medical arena. The OHD and examples of translation code are available at https://github.com/oral-health-and-disease-ontologies/ohd-ontology.

Finally, the OHD only contains a subset of CDT codes. A more complete and formal representation of them and other coding systems relevant to oral health, such as SNO-DDS [43], remains future work.

## Supplementary information

**Additional file 1: Appendix A.** Research questions used in developing the OHD. **Appendix B.** Relate all questions to patient gender and age. SPARQL count by procedure query. **Appendix C.** SPARQL Construct propagating occurrence date. **Appendix D.** SPARQL Query for patients’ age at first dental encounter. **Appendix E.** Query to retrieve the total number of crown restoration procedures. **Appendix F**. Query to retrieve restoration procedures that use resin. **Appendix G.** Query to retrieve tooth surfaces and patients**. Appendix H.** Query to retrieve resin restorations and failures.

## Abbreviations

BFO: Basic Formal Ontology
CARO: Common Anatomy Reference Ontology
CDT: Current Dental Terminology
EHR: Electronic Health Record
FMA: Foundational Model of Anatomy
IAO: Information Artifact Ontology
LSW2: LISP Semantic Web version 2
OBI: Ontology for Biomedical Investigations
OBO Foundry: Open Biological and Biomedical Ontology Foundry
OHD: Oral Health and Disease Ontology
OMRSE: Ontology of Medically Related Social Entities
OWL: Web Ontology Language
RDF: Resource Description Framework
RDF/XML: RDF statements represented using XML syntax
SPARQL: SPARQL Protocol and RDF Query Language
SQL: Structured Query Language
XML: Extensible Markup Language

## Footnotes

1 In this paper, we use the word ‘term’ as a unique natural language expression for a class, instance, or relation in our ontology.

2 http://ontofox.hegroup.org

3 http://purl.org/sig/ont/fma/fma290055 (accessed August 2018)

4 Classes/relations are defined in the OHD unless indicated otherwise.

5 We use Armed Bear Common Lisp (https://abcl.org) for implementation of Common Lisp.

6 https://www.pattersondental.com/eaglesoft (accessed January 2018)

7 LSW provides the means to retrieve the IRI for a term using the syntactic form !<term label>@<ontology label>. In this example,!‘Tooth’@ohd retrieves the IRI for the term ‘Tooth’ (http://purl.obolibrary.org/obo/FMA_12516).

8 https://ontotext.com/products/graphdb/

9 Systemized Nomenclature of Medicine – Clinical Terms, http://www.snomed.org/ (accessed January 2018).

10 The prefix definitions have been excluded. The full query is given in Additional file 1*.*

11 The exact number of billing codes needed depends on the version of ADA codes used.

12 The full query is provided in Additional file 1.

13 The full query and results are provided in Additional file 1.
