# Supplementary material for: Structuring, reuse and analysis of electronic dental data using the Oral Health and Disease Ontology
Source: J Biomed Semantics. 2020 Aug 20;11:8. doi: 10.1186/s13326-020-00222-0 (PMC7439527; doi:10.1186/s13326-020-00222-0)
Supplement: Supplementary file 1 — Additional file 1: Appendix A. Research questions used in developing the OHD. Appendix B. Relate all questions to patient gender and age. SPARQL count by procedure query. Appendix C. SPARQL Construct propagating occurrence date. Appendix D. SPARQL Query for patients’ age at first dental encounter. Appendix E. Query to retrieve the total number of crown restoration procedures. Appendix F. Query to retrieve restoration procedures that use resin. Appendix G. Query to retrieve tooth surfaces and patients. Appendix H. Query to retrieve resin restorations and failures. [file 13326_2020_222_MOESM1_ESM.docx]

1. Research questions used in developing the OHD
2. What is the time from emergence of a tooth to its first restoration (assumes that tooth is observed in its virgin state at least at the first visit)?
3. What is the time span from first restoration on a tooth-to-tooth loss or extraction (assumes that the date of first restoration is known)?
4. What is the time from one restoration to its replacement on the same tooth?
5. Does the time between successive restorations depend on the restorative material such as amalgam and composite?
6. What findings, e.g. caries and fracture, are present on a tooth over time and how do these relate to restorations (e.g., cause for placing the restoration)?
7. How do surfaces of restorations change over time as they are replaced?
8. What is the incidence of root canal treatment by tooth, and how does it relate to the restorative procedures, e.g. as an adverse event?
9. What is the pattern of tooth loss in patients over time?
10. Relate all questions to patient gender and age.SPARQL count by procedure query

The following query was used to retrieve number of instances for each translated procedure:

prefix rdf: <http://www.w3.org/1999/02/22-rdf-syntax-ns#>

prefix rdfs: <http://www.w3.org/2000/01/rdf-schema#>

prefix filling_procedure: <http://purl.obolibrary.org/obo/OHD_0000006>

prefix crown_procedure: <http://purl.obolibrary.org/obo/OHD_0000033>

prefix onlay_procedure: <http://purl.obolibrary.org/obo/OHD_0000134>

prefix inlay_procedure: <http://purl.obolibrary.org/obo/OHD_0000133>

prefix veneer_procedure: <http://purl.obolibrary.org/obo/OHD_0000027>

prefix endodontic_procedure: <http://purl.obolibrary.org/obo/OHD_0000003>

prefix surgical_extraction: <http://purl.obolibrary.org/obo/OHD_0000057>

prefix oral_evaluation: <http://purl.obolibrary.org/obo/OHD_0000197>

select

(count(distinct ?filling) as ?total_fillings)

(count(distinct ?crown) as ?total_crowns)

(count(distinct ?onlay) as ?total_onlays)

(count(distinct ?inlay) as ?total_inlays)

(count(distinct ?veneer) as ?total_veneers)

(count(distinct ?endo) as ?total_endos)

(count(distinct ?extraction) as ?total_extractions)

(count(distinct ?evaluation) as ?total_evaluations)

where {

{?filling rdf:type filling_procedure: .}

union

{?crown rdf:type crown_procedure: .}

union

{?onlay rdf:type onlay_procedure: .}

union

{?inlay rdf:type inlay_procedure: .}

union

{?veneer rdf:type veneer_procedure: .}

union

{?endo rdf:type endodontic_procedure: .}

union

{?extraction rdf:type surgical_extraction: .}

union

{?evaluation rdf:type oral_evaluation: .}

}

1. SPARQL Construct propagating occurrence date

PREFIX occurrence_date: <http://purl.obolibrary.org/obo/OHD_0000015>

PREFIX outpatient_encounter: <http://purl.obolibrary.org/obo/OGMS_0000099>

PREFIX is_part_of: <http://purl.obolibrary.org/obo/BFO_0000050>

PREFIX rdfs: <http://www.w3.org/2000/01/rdf-schema#>

PREFIX process: <http://purl.obolibrary.org/obo/BFO_0000007>

INSERT { ?thing occurrence_date: ?date. }

WHERE

{ ?visit a outpatient_encounter:.

?visit occurrence_date: ?date.

?thing is_part_of: ?visit.

?thing a process:.

optional{?thing occurrence_date: ?existing}.

filter (!bound(?existing))

}

1. SPARQL Query for patients’ age at first dental encounter

Below is the query used to obtain the list of patients, their ages, and dates of their first encounter from the triple store. It is important to note that the encounter dates in the query are being converted into strings. This was necessary in order for the R’s SPARQL library to retrieve sensible results. In the R code, these strings are converted to dates when processing them.

prefix rdf: <http://www.w3.org/1999/02/22-rdf-syntax-ns#>

prefix participates_in: <http://purl.obolibrary.org/obo/BFO_0000056>

prefix health_care_encounter:

<http://purl.obolibrary.org/obo/OGMS_0000096>

prefix dental_patient: <http://purl.obolibrary.org/obo/OHD_0000012>

prefix occurrence_date: <http://purl.obolibrary.org/obo/OHD_0000015>

prefix birth_date: <http://purl.obolibrary.org/obo/OHD_0000050>

select distinct

?patient

(min(?age) as ?min_encounter_age)

(str(min(?encounter_date)) as ?min_encounter_date)

where

{

?patient rdf:type dental_patient: .

?patient participates_in: ?encounter.

?encounter rdf:type health_care_encounter: .

?encounter occurrence_date: ?encounter_date .

?patient birth_date: ?birth_date .

bind(year(?encounter_date)-year(?birth_date) as ?age) .

} group by ?patient

The values from the min_encounter_age field where the used by R’s hist function to produce the **Figure 1**.

1. Query to retrieve the total number of crown restoration procedures

After translating the data source into OWL, loading the OWL into a GraphDB SE (version 8.3) triple store and using GraphDB’s OW2-RL automated reasoner, we retrieved the number of crown restoration procedures by running the following query:

prefix rdf: <http://www.w3.org/1999/02/22-rdf-syntax-ns#>

prefix crown_procedure: <http://purl.obolibrary.org/obo/OHD_0000033>

select (count(distinct ?procedure) as ?total_crowns)

where {

?procedure rdf:type crown_procedure:

}

This query returned 12,636 as the total number of crown procedures.

1. Query to retrieve restoration procedures that use resin

After translating the data source into OWL, loading the OWL into a GraphDB SE (version 8.3) triple store and using GraphDB’s OW2-RL automated reasoner, we retrieved the number of tooth restoration procedures that use resin by running the following query:

prefix rdf: <http://www.w3.org/1999/02/22-rdf-syntax-ns#>

prefix rdfs: <http://www.w3.org/2000/01/rdf-schema#>

prefix resin: <http://purl.obolibrary.org/obo/OHD_0000036>

prefix dental_procedure: <http://purl.obolibrary.org/obo/OHD_0000002>

prefix has_participant: <http://purl.obolibrary.org/obo/BFO_0000057>

prefix asserted_type: <http://purl.obolibrary.org/obo/OHD_0000092>

select ?procedure_name (count(?procedure_type) as ?total)

where {

?material_instance rdf:type resin: .

?procedure_type rdfs:subClassOf dental_procedure: .

?procedure asserted_type: ?procedure_type .

?procedure has_participant: ?material_instance .

?procedure_type rdfs:label ?procedure_name .

} group by ?procedure_name

order by desc(?total)

1. Query to retrieve tooth surfaces and patients

Using the transitive properties of OHD’s ‘is part of’ relation, this query finds patients and their tooth surfaces:

prefix rdf: <http://www.w3.org/1999/02/22-rdf-syntax-ns#>

prefix is_part_of: <http://purl.obolibrary.org/obo/BFO_0000050>

prefix patient: <http://purl.obolibrary.org/obo/OHD_0000012>

prefix tooth_surface:

<http://purl.obolibrary.org/obo/FMA_no_fmaid_Surface_enamel_of_tooth>

select ?surface ?patient

where {

?surface rdf:type tooth_surface: .

?patient rdf:type patient: .

?surface is_part_of: ?patient .

}

1. Query to retrieve resin restorations and failures

SELECT DISTINCT ?patienti ?proci1 ?date1 ?birthdate ?proci2 ?soonest_date2

(MAX(?one_before_date) AS ?previous_visit_date)

(coalesce(?is_male, ?is_female, "unrecorded") AS ?gender)

(coalesce(?is_anterior, ?is_posterior, "dunno") AS ?tooth_type)

(COUNT(DISTINCT ?surfacei) AS ?surface_count)

WHERE

{ { SELECT DISTINCT ?patienti ?proci1 ?date1 ?toothi ?surfacei (MIN(?date2) AS ?soonest_date2)

WHERE

{ ?patienti rdf:type homo_sapiens: .

?toothi rdf:type tooth: ;

is_part_of: ?patienti .

?surfacei rdf:type tooth_surface: ;

is_part_of: ?toothi .

?proci1 rdf:type resin_filling_restoration: .

_:b0 rdf:type tooth_to_be_restored_role: ;

inheres_in: ?toothi .

?proci1 realizes: _:b0 ;

occurrence_date: ?date1 ;

has_participant: ?surfacei ;

later_encounter: ?proci2

{ { { { ?proci2 rdf:type tooth_restoration_procedure: }

UNION

{ ?proci2 rdf:type inlay_restoration: }

}

_:b1 rdf:type tooth_to_be_restored_role: ;

inheres_in: ?toothi .

?proci2 realizes: _:b1 ;

occurrence_date: ?date2 ;

has_participant: ?surfacei

} UNION {

{ { ?proci2 rdf:type crown_restoration: }

UNION

{ ?proci2 rdf:type tooth_extraction: }

UNION

{ ?proci2 rdf:type endodontic_procedure: }

}

_:b2 rdf:type target_of_tooth_procedure: ;

inheres_in: ?toothi .

?proci2 realizes: _:b2 ;

occurrence_date: ?date2

}}}

GROUP BY ?patienti ?toothi ?surfacei ?proci1 ?date1 }

{ { { { ?proci2 rdf:type tooth_restoration_procedure: }

UNION

{ ?proci2 rdf:type inlay_restoration: }}

_:b3 rdf:type tooth_to_be_restored_role: ;

inheres_in: ?toothi .

?proci2 realizes: _:b3 ;

occurrence_date: ?soonest_date2 ;

has_participant: ?surfacei }

UNION

{ { { ?proci2 rdf:type crown_restoration: }

UNION

{ ?proci2 rdf:type tooth_extraction: }

UNION

{ ?proci2 rdf:type endodontic_procedure: }

}

_:b4 rdf:type target_of_tooth_procedure: ;

inheres_in: ?toothi .

?proci2 realizes: _:b4 ;

occurrence_date: ?soonest_date2 }}

?proc_minus_1

next_encounter: ?proci2 ;

occurrence_date: ?one_before_date

OPTIONAL

{ BIND("male" AS ?is_male)

?patienti rdf:type male:

}

OPTIONAL

{ BIND("female" AS ?is_female)

?patienti rdf:type female: }

OPTIONAL

{ BIND("posterior" AS ?is_posterior)

{ { ?toothi rdf:type pre_molar: }

UNION

{ ?toothi rdf:type molar: }}}

OPTIONAL

{ BIND("anterior" AS ?is_anterior)

{ { ?toothi rdf:type canine: }

UNION

{ ?toothi rdf:type incisor: }}}

OPTIONAL

{ ?patienti birth_date: ?birthdate }

}

GROUP BY ?patienti ?proci1 ?date1 ?birthdate ?proci2

?soonest_date2 ?is_male ?is_female ?is_anterior ?is_posterior
